# A Most Irregular Threat: Old Gas Regulators Can Present Mercury Exposure

**Published:** 2006-06

**Authors:** Ernie Hood

Residential gas regulators reduce the pressure of gas from main feeder lines to usable levels for household pipes. Gas regulators built before 1961 were commonly located within dwellings and incorporated a component that contained, on average, 136 grams (about 2 teaspoons) of elemental mercury. Newer units do not contain mercury, but as a group of Chicago-area researchers report, careless replacement of the older units can result in potentially hazardous mercury contamination **[*EHP* 114:848–852; Hryhorczuk et al.]**.

The authors describe how this newly identified source of residential mercury exposure came to light in 2000, when a suburban Chicago family discovered a pool of the silvery element on the floor of their basement workroom six weeks after a gas company contractor removed and replaced their gas regulator and meter. An investigation showed that the air in the house contained elevated levels of mercury vapor, and the father and the 9-year-old son, who spent more time in the basement area than the mother, had blood and urine mercury levels above recommended background limits. Short-term exposure to high levels of metallic mercury vapors can cause lung damage, nausea, vomiting, diarrhea, elevated blood pressure or heart rate, skin rash, and eye irritation.

Fortunately, ventilation and remediation of the home brought mercury concentrations in the home’s air down to safe levels, and several weeks after exposure ceased, the father’s and son’s blood and urine mercury levels returned to normal background ranges. Neither ever manifested overt clinical signs of mercury poisoning.

This case and a cluster of similar cases quickly caught the attention of area authorities. Ultimately, two area gas companies were required to conduct inspections in 361,000 homes where their employees or subcontractors had replaced old gas regulators. Free urine mercury screenings were offered to concerned residents.

Of the 301,000 homes inspected by one of the companies, 1,308 (0.43%) were found to be contaminated, and 1,033 were remediated. Of 60,000 homes screened by the other company, 55 (0.09%) were found to be contaminated and were remediated. The risk was considerably higher in homes where the equipment had been replaced by one particular subcontractor—of 120 homes screened, 20 (16.7%) were found to have been contaminated by mercury.

Of the 625 residents who elected to undergo urine mercury screenings, 9 (1.4%) had positive bioassays, defined as a 24-hour urine mercury concentration equal to or higher than 10 micrograms per liter. Although none of the subjects showed overt symptoms of mercury poisoning, as the authors point out, the screenings were not designed to detect subclinical effects of mercury exposure.

Interestingly, positive urine mercury in residents was more strongly associated with elevated air mercury concentrations on the first floor of the homes than with elevated basement air levels, even though the air concentrations were considerably higher in basements, where the spills typically occurred. The authors attribute this result to the simple fact that people generally spend less time in their basements than in the living quarters aboveground.

The Chicago episode not only revealed a previously unidentified environmental exposure hazard but also provided valuable lessons on how to respond quickly and efficiently. As the authors note, gas companies and their subcontractors, clinicians, public health and environmental officials, and residents all need to be aware of the potential for contamination in older homes or other buildings where mercury-containing gas regulators have been replaced in the past, or where they may still exist.

## Figures and Tables

**Figure:**
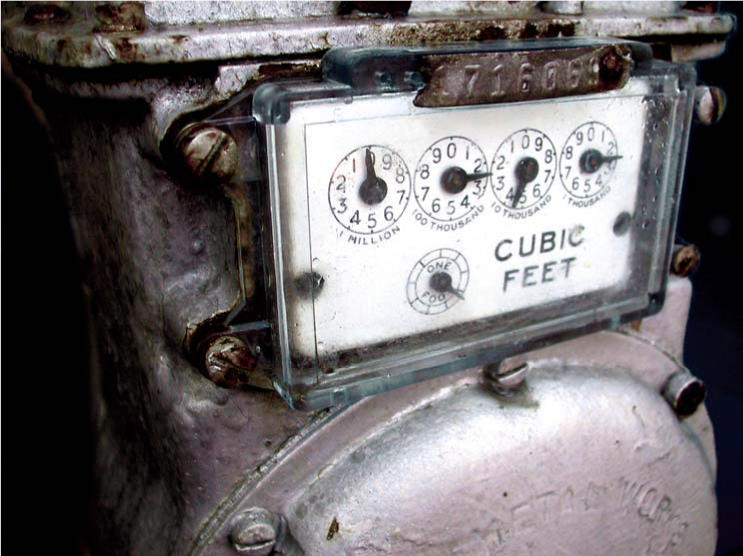
Old equipment poses new problem Old gas regulators that contain mercury can present a potential health hazard to residents if care is not taken when they are replaced.

